# Drug-eluting stent in diabetes

**DOI:** 10.1590/S1516-31802007000500001

**Published:** 2007-09-02

Long-term evaluation of cardiovascular risk among diabetic patients has shown that the incidence of death due to myocardial infarction among these patients is 20%, whereas among nondiabetic patients it is only 3.5%. Such observations haveled to classifying diabetes as a risk that is equivalent to established coronary artery disease.^1^ Among patients with established coronary artery disease, the presence of diabetes has been found to increase the five-year mortality rate to more than twice what is seen for nondiabetic patients.^2^ The long-term outcome for patients with diabetes following revascularization is also adverse. Increased postprocedure mortality among angioplasty patients with diabetes has been documented (3.2% *versus* 0.5%).^3^ Diabetes also confers a worse long-term outcome among patients undergoing bypass surgery. The Society of Thoracic Surgeons’ database shows that the surgical risk for diabetic patients is greater than for nondiabetic patients: 3.7% *versus* 2.7%, with greater risk for those requiring insulin.^4^

Diabetes gives rise to a number of metabolic disturbances that result in progression of atherosclerosis and occurrence of cardiac events. The presence of hyperglycemia, abnormal lipid metabolism and insulin resistance, together with occurrences of hypertension, all result in acceleration of the atherosclerotic process. Increased oxidative stress results from decreased nitric oxide production, which is caused by altered endothelial cell function due to hyperglycemia.^5^ Release of free fatty acids from adipose tissue due to insulin resistance leads to oxidative stress and decreased nitric oxide production.^6^ Increased endothelin production in diabetic patients also results in vasoconstriction. Platelet function is also altered in diabetes cases.^7^ Lipid abnormalities may result in elevated triglyceride levels with decreased high density lipoprotein (HDL) levels.^8^ Hypertension may be associated with diabetes and renal dysfunction. Rapid progression of atherosclerosis and a greater tendency towards unstable platelets result in cardiac events.

Whenever a promising study does not produce the intended solution, there is a tendency towards focusing it on a subgroup of more fragile patients, who are therefore more likely to respond well to the treatment in question. This happens particularly with studies on diabetes because of the destructive power of this disease in relation to vascular capability. Whereas physiopathological reasoning is elegant, its verification is shaky. Thus, despite many studies of high methodological quality looking for the best evidence regarding the effectiveness of drug-eluting stents in coronary diseases among subgroups of diabetic patients,^9–26^ there have not been any data from randomized controlled trials that could be used in meta-analyses. This seems to us to be a deliberate omission of data without statistical significance, although some studies make reference to diabetes in their presentations of results. On the other hand, these trials have frequently presented separate comments referring to diabetic patients.

A recently published systematic review^27^ compared drug-eluting stents and bare-metal stents, and additionally compared sirolimus-eluting stents *versus* paclitaxel-eluting stents. Five aims were defined for the study, and stent type in relation to diabetes was not among these aims, even though that review cited three studies^28–30^ in which 100% of the patients were diabetic. The discussion in that review makes contradictory statements regarding diabetic patients, while seeming to take a position in favor of bare-metal stents.

To date, there has not been any evidence that drug-eluting stents are preferable for treating diabetic patients with coronary disease. The data omissions in the published studies make this statement clear. As can be seen from the main studies, there have not been any data supporting the effectiveness of drug-eluting stents and consequently their use as the preferential treatment for coronary disease in diabetic patients. Many revascularization results have been given without specifying whether the revascularization was surgical or percutaneous. This lack of precision in defining outcomes has caused many misleading conclusions in the medical literature in this field.

## References

[1] Stein B, Weintraub WS, Gebhart SP (1995). Influence of diabetes mellitus on early and late outcome after percutaneous transluminal coronary angioplasty. Circulation.

[2] Barzilay JI, Kronmal RA, Bittner V, Eaker E, Evans C, Foster ED (1994). Coronary artery disease and coronary artery bypass grafting in diabetic patients aged > or = 65 years (report from the Coronary Artery Surgery Study [CASS] Registry). Am J Cardiol.

[3] Silva JA, Escobar A, Collins TJ, Ramee SR, White CJ (1995). Unstable angina. A comparison of angioscopic findings between diabetic and nondiabetic patients. Circulation.

[4] Moreno PR, Murcia AM, Palacios IF (2000). Coronary composition and macrophage infiltration in atherectomy specimens from patients with diabetes mellitus. Circulation.

[5] Pickup JC, Chusney GD, Thomas SM, Burt D (2000). Plasma interleukin-6, tumour necrosis factor alpha and blood cytokine production in type 2 diabetes. Life Sci.

[6] Marx N, Imhof A, Froehlich J (2003). Effect of rosiglitazone treatment on soluble CD40L in patients with type 2 diabetes and coronary artery disease. Circulation.

[7] Blüher M, Unger R, Rassoul F, Richter V, Paschke R (2002). Relation between glycaemic control, hyperinsulinaemia and plasma concentrations of soluble adhesion molecules in patients with impaired glucose tolerance or Type II diabetes. Diabetologia.

[8] Morrison DA, Sethi G, Sacks J (2002). Percutaneous coronary intervention versus coronary bypass graft surgery for patients with medically refractory myocardial ischemia and risk factors for adverse outcomes with bypass: The VA AWESOME multicenter registry: comparison with the randomized clinical trial. J Am Coll Cardiol.

[9] Moses JW, Leon MB, Popma JJ (2003). Sirolimus-eluting stents versus standard stents in patients with stenosis in a native coronary artery. N Engl J Med.

[10] Schampaert E, Cohen EA, Schlüter M (2004). The Canadian study of the sirolimus-eluting stent in the treatment of patients with long de novo lesions in small native coronary arteries (C-SIRIUS). J Am Coll Cardiol.

[11] Schofer J, Schlüter M, Gershlick AH (2003). Sirolimus-eluting stents for treatment of patients with long atherosclerotic lesions in small coronary arteries: double-blind, randomised controlled trial (E-SIRIUS). Lancet.

[12] Morice MC, Serruys PW, Sousa JE (2002). A randomized comparison of a sirolimus-eluting stent with a standard stent for coronary revascularization. N Engl J Med.

[13] Park SJ, Shim WH, Ho DS (2003). A paclitaxel-eluting stent for the prevention of coronary restenosis. N Engl J Med.

[14] Gershlick A, De Scheerder I, Chevalier B (2004). Inhibition of restenosis with a paclitaxel-eluting, polymer-free coronary stent: the European evaLUation of pacliTaxel Eluting Stent (ELUTES) trial. Circulation.

[15] Lansky AJ, Costa RA, Mintz GS (2004). Non-polymer-based paclitaxel-coated coronary stents for the treatment of patients with de novo coronary lesions: angiographic follow-up of the DELIVER clinical trial. Circulation.

[16] Grube E, Silber S, Hauptmann KE (2003). TAXUS I: six- and twelve-month results from a randomized, double-blind trial on a slow-release paclitaxel-eluting stent for de novo coronary lesions. Circulation.

[17] Colombo A, Drzewiecki J, Banning A (2003). Randomized study to assess the effectiveness of slow- and moderate-release polymer-based paclitaxel-eluting stents for coronary artery lesions. Circulation.

[18] Stone GW, Ellis SG, Cox DA (2004). A polymer-based, paclitaxel-eluting stent in patients with coronary artery disease. N Engl J Med.

[19] Grube E Taxus VI: 9 months results. Insight into diabetics.

[20] Grubeng L (2004). TAXUS VI: Paclitaxel-Eluting Stents for the Treatment of Longer Lesions – Focus on Diabetes.

[21] Five-year clinical and functional outcome comparing bypass surgery and angioplasty in patients with multivessel coronary disease (1997). A multicenter randomized trial. Writing Group for the Bypass Angioplasty Revascularization Investigation (BARI) Investigators. JAMA.

[22] King SB, Kosinski AS, Guyton RA, Lembo NJ, Weintraub WS (2000). Eight-year mortality in the Emory Angioplasty versus Surgery Trial (EAST). J Am Coll Cardiol.

[23] Serruys PW, Ong AT, van Herwerden LA (2005). Five-year outcomes after coronary stenting versus bypass surgery for the treatment of multivessel disease: the final analysis of the Arterial Revascularization Therapies Study (ARTS) randomized trial. J Am Coll Cardiol.

[24] Zhang Z, Mahoney EM, Stables RH (2003). Disease-specific health status after stent-assisted percutaneous coronary intervention and coronary artery bypass surgery: one-year results from the Stent or Surgery trial. Circulation.

[25] Breeman A, Bertrand ME, Ottervanger JP (2006). Diabetes does not influence treatment decisions regarding revascularization in patients with stable coronary artery disease. Diabetes Care.

[26] Ben-Gal Y, Moshkovitz Y, Nesher N (2006). Drug-eluting stents versus coronary artery bypass grafting in patients with diabetes mellitus. Ann Thorac Surg.

[27] Stettler C, Wandel S, Allemann S (2007). Outcomes associated with drug-eluting and bare-metal stents: a collaborative network meta-analysis. Lancet.

[28] Sabaté M, Jiménez-Quevedo P, Angiolillo DJ (2005). Randomized comparison of sirolimus-eluting stent versus standard stent for percutaneous coronary revascularization in diabetics patients: the diabetes and sirolimus-eluting stent (DIABETES) trial. Circulation.

[29] Chan C, Zambahari R, Kaul U, Cohen SA, Buchbinder M (2005). Outcomes in diabetics patients with multivessel disease and long lesions: results from the DECODE STUDY. Am J Cardiol.

[30] Baumgart D, Klauss V, Baer F (2006). 1-year results of the SCORPIUS Trial - a German multicenter investigation of the effectiveness of sirolimus-eluting stents in diabetic patients.

